# Syrian refugee children in Turkey and coronavirus disease 2019: A close-up view

**DOI:** 10.7189/jogh.12.03007

**Published:** 2022-03-05

**Authors:** Fahad Ahmed

**Affiliations:** 1 Department of Public Health, Faculty of Medicine, Ankara Yıldırım Beyazıt University, Ankara, Turkey; 2 Research for Health in Conflict (R4HC-MENA) Programme’, Department of Pediatric Oncology, Hacettepe University, Ankara, Turkey

At the beginning of the COVID-19 pandemic in Turkey on March 11, 2020 [1] Turkey was hosting more than 3.58 million Syrian refugees including 1.5 million children aged less than 15 years and 98% were living outside the camps [2]. The health, economic, and social vulnerability of the refugees have been a major concern since the beginning of the COVID-19 pandemic but the real impact of the pandemic is yet to be measured especially among the most vulnerable sub-groups like refugee children.

Engaging refugee children in research is a very sensitive issue. This narrative is based on the author's observations and informal talks with Syrian refugee children aged 7-12 years and their parents while meeting in the mosques, streets, and markets, on several occasions. These families are living in the neighborhood and have known the author for four years. This narrative aims to highlight the adverse effects beyond the infection that this pandemic has had on the lives of refugee children. It is hoped that it will give some clues and serve as a baseline for in-depth research as well as aid international humanitarian aid organizations to focus on the emerging needs of these children.

The COVID-19 pandemic has affected refugee populations disproportionately around the world [3]. Turkey is no exception, having been severely affected by this pandemic and facing a chronic refugee crisis [4]. The pandemic fuelled a gap between limited resources and unlimited needs in the national health care system, which pushed the Turkish Ministry of Health to adopt a pandemic-oriented approach. The national health care system was transformed for prioritizing COVID-19 patient care and the protection of health care workers’ well-being. Even though this approach was successful in preventing the devastating consequence of the pandemic, it resulted in reduced access to routine care and interruption of ongoing health care services for citizens as well as refugees [5]. Access to some outpatient treatments like skin, dental and ophthalmological care that was given to the refugee children before the pandemic were reduced. However, access to routine immunization and emergency services remained unaffected. The Government of Turkey has provided assurances that refugees will have equal access to COVID-19 diagnosis, treatment, and vaccination par with the host community [6]. However, the language barrier is a potential factor for low access level [7]. Most of the guidelines and health information materials at public places and on media are in the Turkish language which could limit access to the COVID-19 information for non-Turkish speakers.

Quality education is the foundation of health and well-being. The evidence shows that early childhood education has the potential for preventing disease and promoting health in adulthood [8]. The lockdowns in response to the pandemic have interrupted conventional schooling in Turkey. Like many other countries, the COVID-19 lockdowns have shifted the traditional mode of teaching into online education in Turkey through a distance learning “Education Information Network”. This online education is a challenging experience for both teachers and students [9]. Although Syrian students are included in the national education system, but the pandemic made them more underprivileged compared to host peers. One of the major difficulties for Syrian refugee children is having limited access to a TV, computer, smartphone, or the internet. Some of these children and their parents have never used these technologies. Moreover, most households have with multiple school-aged children, and it is extremely difficult for all children in one family to benefit from distance learning. Less eagerness to learn and limited help from parents is another obstacle, it is a common observation that Syrian parents are not fluent in speaking Turkish and are not able to understand the content of lessons and thus these children do not get enough support from parents. Notably, the Syrian children acknowledged that Turkish teachers were helpful and sensitive to their needs. The United Nations Children’s Fund also supports the establishment of mobile centres helping children without computer and/or internet access to continue their education [10]. However, these initiatives are not yet able to can’t reach all refugee children, considering their high number. The Turkish education system is based on equality, whereby host and refugee students are treated equally. Despite this, after the re-opening of schools, increased resources and funding is crucial to maintain equity in education access, whereby refugee students can cover any education loss and not lag behind their host peers. In the long run, education systems must be transformed and built to be resilient and inclusive, so that generational catastrophe will be avoided [11].

Nutritional issues of children are another consideration during the COVID-19 pandemic [12]. To overcome nutritional challenges various non-governmental organizations and local governments provided ration support (lentils, rice, flour, tea, sugar, and cooking oil but lacking protein-based like eggs, meat, and milk) to Syrian families. This is often insufficient and refugee children also collect half-rotten vegetables and fruits from local grocers. The lack of proper nutrition among children is apparent as loss of weight compared to pre-pandemic periods, easy fractures, and poor wound healing. Syrian girls are concerned with hair and height issues and wish to buy shampoos and nutritional supplements shown in commercials. The monthly cash allowance (155 TRY, about US$18 monthly per family member) via the Emergency Social Safety Net Programme [13] is just about sufficient to pay house rents and utility bills, but insufficient to buy healthy food. The Turkish government has designated a national fund assistance program in response to the economic impact of the pandemic [14], that targets only vulnerable host communities, and is ineligible for refugees. However, some charitable organizations, are actively delivering rations assistance to Syrian refugees and vulnerable host communities. Eventually to battle against malnutrition more financial and food assistance is crucial.

Most of Syrian refugees in Turkey do not have stable jobs [15], with high poverty rates presenting a serious problem for them. The national relative poverty rate for children excluding refugees was 22.8% in 2019 and is higher than that for the general population. [16]. Considering available data and estimates of the impact of the COVID-19 pandemic on global poverty [17], it can be assumed that poverty is becoming a bigger problem universally among refugees. Lockdown and other restrictions imposed by the government during the pandemic were applied equally for both the host community and refugees in Turkey, yet these lockdown measures revealed more hardships for refugee communities, especially with regard to the caused restrictions on appropriate earnings. For children, the financial challenges were further exaggerated as the number of philanthropists, especially elderly host people, stayed at home or prayed at home due to the COVID-19 precautions. This resulted in a considerable reduction in the charity money provided to these refugee children. Consequently, some refugee children found jobs like helpers in local shops, domestic work, and waste picking. This raises concern as these children may not be able to return to school post-pandemic. Adolescent girls are disproportionately affected by this economic stress, and are more likely to marry earlier, and potentially increase gender inequalities in the post-pandemic era. Turkey has committed to eradicating gender inequalities and child labour but this pandemic might become an obstacle to accelerating its efforts. Measures should be put in place to get every child back into school.

**Figure:**
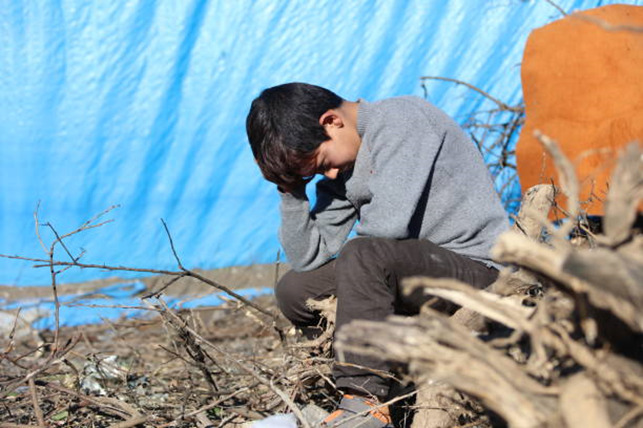
Photo: Source: istock photo 911497078.

The effects of COVID-19 on the mental health of global populations are widely recognized [18]. While the refugees are already struggling with the trauma of displacement and readjustment the pandemic associated stress has a more negative impact on their mental health. Staying at home, loss of earnings, and social isolation have increased in-home family conflicts. This is also risking the children's psychological and behavioural development. Under the various strains, these children are becoming more aggressive and stubborn compared to the pre-pandemic period. Lack of indoor play equipment and toys forced them to spend more time in the neighbourhood streets. Their unusual activities and disturbing noise while playing outside created undesired and harsh reactions from the host neighbours who were already struggling themselves with the challenges of lockdown. Xenophobia is becoming more apparent during this pandemic; Syrian refugees are blamed for unemployment and financial crisis [19]. Undoubtedly there is political will for the social, cultural, and economic integration of Syrian refugees. Despite this, the economic strain in the society fuels hostility and possibly threatens the efforts of integration.

Corona-phobia is higher among these children. They know that they could transmit it, therefore, increasingly anxious that if they or their parents become sick it could lead to isolation and separation from parents. Moreover, these children are more compliant with coronavirus legislation and fear that violation of rules could result in deportations. They fear any person in a police-like uniform and try to run away or hide while playing in the streets. The economic strain is an obstacle to buying face masks, sanitizers, and disinfectants. They either use the same mask for many days or share masks with siblings. As the price of hand sanitizer is not affordable they either don’t use it or rely on publicly placed sanitizer dispensers. Although clear evidence is lacking, but poverty, malnutrition, and poor infrastructure are likely put additional weight on an increased risk of COVID-19 infection, morbidity, and mortality among refugees.

Malnutrition, lack of education, poor physical and mental health has the potential for long-term consequence on these children. More evidence-based studies are needed to unveil the burden of the COVID-19 pandemic on Syrian refugee children. This pandemic has seized the capacities of all countries to respond to the needs of its citizens and for countries hosting refugee populations in large numbers, it has been twice as difficult. Turkey is known for its generosity toward refugees and is committed to providing basic services. However, the battle against inequalities created by this pandemic cannot be won without global cooperation and shared responsibility. One particular issue is the decline in humanitarian aid to the major relief programs [20], due to the detrimental economic effects of the pandemic. The future of millions of Syrian refugee children will be in danger if the assistance is withdrawn or fall shorter compared to the actual needs. Noteworthy, the current refugee policy of Turkey is supportive towards Syrian [21], there is a political will for refugee protection and solutions of refugees’ issues. In this context, beyond foreign aid, the economic and political stability within the Turkish society is key for the protection of refugee populations by the provision of financial assistance, adequate health care, and education, while preventing xenophobia and mitigating the impact of the COVID-19 crisis.
